# Patient-Specific Computational Framework to Spatiotemporally Forecast Treatment Response and Identify Biologically Informed Radiotherapy Targets in High-Grade Glioma

**DOI:** 10.1200/CCI-25-00336

**Published:** 2026-09-24

**Authors:** Sophia Ty, Jeanne Kowalski, Bikash Panthi, Maguy Farhat, Holly Langshaw, Mihir D. Shanker, Wasif Talpur, Sara Thrower, Jodi Goldman, Calliope Custer, Thomas E. Yankeelov, Caroline Chung, David A. Hormuth

**Affiliations:** ^1^ Oden Institute for Computational Engineering and Sciences, The University of Texas at Austin, Austin, TX; ^2^ UT Austin Cancer Research Institute, The University of Texas at Austin, Austin, TX; ^3^ Department of Medicine, The University of Texas at Austin, Austin, TX; ^4^ Department of Radiation Oncology, The M.D. Anderson Cancer Center, Houston, TX; ^5^ Faculty of Medicine, The University of Queensland, Brisbane, Queensland, Australia; ^6^ Department of Population Health, The University of Texas at Austin, Austin, TX; ^7^ Department of Biomedical Engineering, The University of Texas at Austin, Austin, TX; ^8^ Department of Diagnostic Medicine, The University of Texas at Austin, Austin, TX; ^9^ Department of Imaging Physics, The M.D. Anderson Cancer Center, Houston, TX

## Abstract

**Purpose:**

High-grade gliomas exhibit substantial spatial and temporal heterogeneity, posing a challenge for radiotherapy (RT) target definition. Adaptive RT, which prospectively modifies treatment plans, may benefit from early identification of intratumoral regions likely to exhibit differential treatment response. Apparent diffusion coefficient (*ADC*), a marker associated with tumor cellularity and aggressiveness, is a promising imaging biomarker of RT response. We present a patient-specific computational framework that predicts treatment response and identifies candidate adaptive RT targets from anticipated spatiotemporal patterns of *ADC* changes (Δ*ADC*).

**Methods:**

Using longitudinal multiparametric magnetic resonance imaging (MRI) from 21 patients, the mathematical model was calibrated to spatiotemporally forecast tumor cell density immediately after and 1-month post-RT. Predictions of tumor cell density were converted to *ADC* maps. Δ*ADC* from baseline was analyzed using Pareto tail analysis. The lower Δ*ADC* distribution tail was used to classify tumor voxels as regions of increased diffusion restriction (IDR) or decreased/maintained diffusion restriction (DMDR). We compared agreement in categories between measured and predicted Δ*ADC* from MRI data. Classification performance was assessed using receiver operating characteristic (ROC) analysis across four scenarios combining two time points and two image inclusion strategies (± nonenhancing tumor volume).

**Results:**

The framework achieved a median ROC AUC of 0.88 (0.80-0.93), indicating strong performance in distinguishing IDR from DMDR regions. No significant differences were observed between forecasting time points (*P* value: .08) or image inclusion strategies (*P* value: .50).

**Conclusion:**

This framework can delineate IDR and DMDR regions, offering a potential strategy for prospectively defining candidate biologically informed adaptive RT targets. Future studies are needed to evaluate their role in adaptive RT planning.

## Introduction

High-grade gliomas (HGGs) are difficult to treat given their infiltrative nature and propensity to rapidly recur after first-line therapy.^1^ This outcome is, in part, an effect of the challenges in targeting refractory tumor areas during radiotherapy (RT).^2^ For decades, HGG treatment has used uniform radiation delivery, overlooking variations in biological behavior within a tumor observed in biomedical images.^2,3^ Current therapeutic protocols, determined by the population-average response seen in clinical trials, also fail to account for differences in clinical responses among patients with HGG.^4^

**Context**

**Key Objective**
To account for biological variations when defining candidate adaptive radiotherapy (RT) targets in high-grade glioma.
**Knowledge Generated**
Integrating mathematical tumor growth forecasting with data-driven analysis of diffusion restriction allows (1) prediction of patient-specific tumor progression and treatment response, and (2) identification of intratumoral biological heterogeneity through changes in apparent diffusion coefficient (*ADC*).
**Relevance *(N. Wood)***
This work presents a patient-specific approach that combines tumor modeling with diffusion magnetic resonance imaging to predict treatment response and map intratumoral heterogeneity. By moving beyond margin-based target definition, it offers a strategy to identify biologically informed regions for adaptive RT and support more personalized treatment in HGG.*
*Relevance section written by *JCO Clinical Cancer Informatics* Associate Editor Nicole Wood, DO.

Adaptive RT approaches that account for disease responsiveness have since emerged to improve RT.^5^ These techniques, which use tailored dose maps based on tumor alteration patterns, surpass historical attempts to modify RT regimens based on tumor shape or deformation.^4-6^ Several studies have already demonstrated their ability to improve HGG survival outcomes.^6,7^ However, these adaptive RT schemes have yet to be broadly implemented in the clinic. A major barrier to this goal is the lack of reliable methods (and consensus^8,9^) for defining candidate *biologically informed* RT targets that may benefit from dose boosts or adaptive dose plans.

Other attempts to define adaptive RT targets expand tumor boundaries visible in preoperative and postoperative magnetic resonance imaging (MRI) scans and MRI fusion images.^10-12^ These studies use different margin sizes, highlighting the dissonance in defining RT targets.^9-16^ Additionally, selecting tumor boundaries and nearby tissue disregards intratumoral heterogeneity. A recent systematic review found that progression and recurrence occur within previously irradiated volumes for 69%-93% of patients, whereas failures near tumor boundaries occur for up to 12.5% of patients.^17^ This aligns with previous studies reporting margin recurrence for approximately 10% of patients.^18-20^ A study by Paulsson et al^21^ also demonstrated that varying margin expansions between 5 and 20 mm did not lead to significant differences in failure patterns. Thus, alternate approaches for identifying candidate biologically informed targets within the tumor are needed.

A more quantitative approach for describing treatment-induced biological changes in the tumor is based on functional diffusion mapping (fDM). fDM quantifies voxel-wise changes in the apparent diffusion coefficient (*ADC*; a measure of water diffusion in tissue) using diffusion-weighted MRI (DWI).^22^ A substantial body of experimental and clinical evidence demonstrates an inverse relationship between *ADC* and tumor cellularity, with increased cell density restricting water diffusion and resulting in lower *ADC* values, highlighting *ADC*'s potential as an imaging biomarker of treatment response.^23^ Consequently, diffusion restriction is incorporated into clinical MRI assessment as part of multiparametric evaluation of tumor aggressiveness and therapeutic response.^24^

Although diffusion metrics are influenced by biological and treatment-related factors such as cellular density, necrosis, edema, vascular permeability, extracellular matrix composition, and therapy-induced changes, increased cell density has been associated with lower *ADC* values across tumor types, including gliomas. In HGG, low *ADC* also correlates with higher grade disease and poorer prognosis, supporting its use as a clinically relevant imaging feature.^25-27^For this reason, we propose a mapping strategy akin to fDM using changes in *ADC* as a probabilistic imaging biomarker that can be adjusted for local protocols and dose-escalation preferences—such as targeting different quantiles of changes in *ADC*—at a given institution.

We have previously developed a mathematical model of tumor growth and response that predicts changes in tumor cell density (and, therefore, *ADC*) during RT.^28,29^ We now extend this framework to include a statistical, data-driven approach to classify tumor voxels based on changes in *ADC*.

## Methods

### Description of HGG Cohort

Our cohort includes 21 patients with biopsy-confirmed HGGs treated and consented to participate in a single-arm prospective clinical trial (ClinicalTrials.gov identifier: NCT04771806)^30^ approved by the institutional review board at M.D. Anderson Cancer Center. Patients had either gross total or partial tumor resection and were subsequently treated with chemoradiation following the Stupp Protocol (concomitant chemotherapy using temozolomide during 6 weeks of radiation therapy).^31^ Clinical characteristics of our cohort are summarized in the Data Supplement (Table S1).

MRI scans were acquired throughout HGG management to report tumor cell density and disease extent (protocol details in Data Supplement). Imaging was performed postoperatively before RT (baseline), weekly during RT, and at standard-of-care follow-up visits (eg, 1-3 months) as part of the trial.^30^ For this analysis, we used MRI from baseline through 1-month post-RT (*RT*_*1 month*_). Each session included DWI and *T*_1_-weighted imaging; gadolinium-enhanced *T*_1_-weighted images were obtained at baseline, in the middle of RT (*RT*_*mid*_), and during follow-ups. Image processing, registration, and segmentation are described in the Data Supplement and prior studies.^28,29^ Because *ADC* derived from DWI is central to our framework, we assessed its repeatability using Bland-Altman analysis at both volume and voxel levels in healthy-appearing brain tissue. Mean differences ranged from –2.12 × 10^−4^ to 1.47 × 10^−4^ mm^2^/s, indicating high measurement reliability (Data Supplement, Figs S1 and S2).

### Patient-Specific Mathematical Modeling of HGG Growth and Treatment Response

Tumor cell volume fraction was calculated within the enhancing tumor region from the *ADC* to estimate tumor cell density as previously described^28,32-37^ using Equation 1:

$$\begin{array}{c} \phi_{T}\left(\bar{x},t\right)=\left(\frac{{ADC}_{w}-ADC\left(\bar{x},t\right)}{{ADC}_{w}-{ADC}_{\min}}\right), \end{array}$$ (1)

where $\phi_{T}\left(\bar{x},t\right)$ is the tumor volume fraction at the 3D position $\bar{x}$ and time *t*, *ADC*_*w*_ is the *ADC* of free water measured at 32°C,^38^ and *ADC*_*min*_ is the minimum value of the measured *ADC.* Tumor cell volume fraction at nonenhancing regions was fixed to 0.16.^39^

Our mathematical model is a two-species reaction-diffusion equation describing tumor cell invasion, proliferation, and treatment response across enhancing and nonenhancing tumor regions (Eqs 4-7, Data Supplement).^28^ Radiation and chemotherapy response is modeled as an immediate reduction in tumor volume. Model details including its calibration, evaluation, boundary conditions, and parameter values have been previously published^29^ with salient details outlined in the Data Supplement. Tumor forecasts were generated for each patient at *RT*_*end*_ and *RT*_*1 month*_ to obtain patient-specific predictions of $\phi_{i}$ that were converted to *ADC* using Equation 1.^29^

### Data-Driven Spatial Mapping of RT Targets

Voxel-wise changes in *ADC* were calculated between a future image time point (ie, *RT*_*end*_ or *RT*_*1 month*_) and the baseline image for both measured and model-predicted *ADC* maps. Comparisons of Δ*ADC* between the initial (*t*_0_) and final (*t*_*f*_) time points were calculated as follows:

$$\begin{array}{c} {\Delta ADC}_{measured}=\text{Measured}\;{ADC}_{tf}-{ADC}_{t0} \end{array}$$ (2)

$$\begin{array}{c} {\Delta ADC}_{\text{predicted}}=\text{Predicted}\;{ADC}_{tf}-{ADC}_{t0}, \end{array}$$ (3)

where Δ*ADC*_*measured*_ and Δ*ADC*_*predicted*_ are the observed and model-predicted maps of voxel-wise changes in *ADC*, respectively. Some studies have observed an inverse association between *ADC* and tumor cellularity.^23-27^ In line with these reports, decreases in *ADC* (ie, Δ*ADC* <0) were considered suggestive of increased tumor cell density, whereas increases in *ADC* (ie, Δ*ADC* >0) were considered suggestive of decreased tumor cell density. No filtering was performed to exclude nontumor contributions to low *ADC*.

For each patient, we estimated an empirical cumulative distribution function fit of the voxel-level changes in *ADC* and conducted piecewise evaluation of the distribution using a generalized pareto tail distribution analysis. A portion of the tumor was identified as a candidate imaging-defined RT target from the lower tail of the Δ*ADC* distribution using an a priori assigned cumulative probability cutoff. The Δ*ADC* associated with the assigned probability cutoff was used as the threshold to label tumor voxels as regions with increased diffusion restriction (IDR) or regions with decreased/maintained diffusion restriction (DMDR) following treatment. Voxels were categorized as DMDR if its Δ*ADC* is above the threshold and IDR if the value is less than or equal to the threshold. We applied this workflow for the ground truth and model-predicted Δ*ADC* maps independently. We compared tumor region categories between ground truth and model-predicted data to assess our computational framework's ability to forecast and delineate candidate RT targets. This analysis was iteratively implemented using different lower tail cumulative probability (LTCP) cutoffs at 20%, 25%, 30%, 35%, and 40% in MATLAB (R2023a) using *ecdf*, *paretotails*, and *confusionmatrix*. Figures 1A-1E summarizes the statistical workflow performed on the Δ*ADC* maps from the observed data and predicted outputs of our mathematical model.

**Fig 1.**
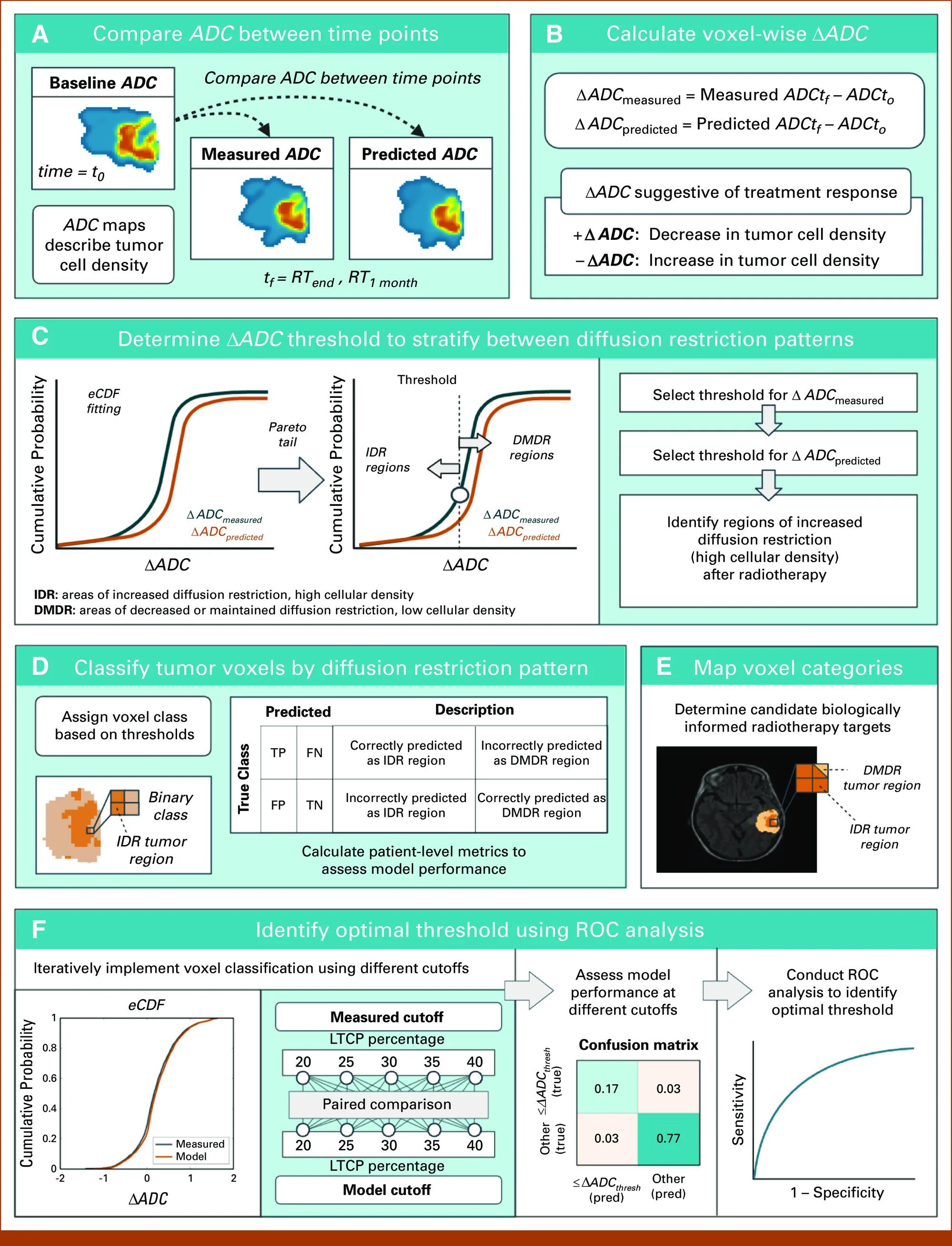
Summary of the workflow for identifying candidate biologically informed HGG targets for radiotherapy. (A) Baseline *ADC* maps acquired during treatment planning are compared with the measured and predicted *ADC* maps at the end of radiotherapy (*RT*_*end*_) and 1-month follow-up (*RT*_*1 month*_). (B) Changes in tumor *ADC* (Δ*ADC*) following radiotherapy are calculated by taking the voxel-wise difference between the *ADC* values at the final time point (*t*_*f*_) and the initial time point (*t*_0_) for measured and predicted data. Voxels with positive changes in *ADC* (+Δ*ADC*) are considered suggestive of decreased tumor cell density, whereas negative changes in *ADC* (−Δ*ADC*) are considered suggestive of increased tumor cell density. (C) Empirical cumulative distribution function fits of the measured and predicted Δ*ADC* were obtained. Generalized Pareto tail analysis was used to identify tumor voxels within and outside a given Δ*ADC* threshold associated with independently assigned lower tail cumulative probability cutoffs for Δ*ADC*_*measured*_ and Δ*ADC*_*predicted*_. (D) Each voxel was classified as either an IDR (areas of IDR) or DMDR (areas of decreased/maintained diffusion restriction) region. Patient-level metrics were calculated using contingency tables to assess model performance with IDR regions as the positive class. (E) Candidate biologically informed radiotherapy targets were spatially mapped to the anatomical MRI to visualize tumor regions classified as DMDR or IDR shown in light orange and dark orange, respectively. (F) Pareto tail analysis was conducted at five different lower tail cumulative probability cutoffs (20%, 25%, 30%, 35%, 40%) for both measured and model data. Voxel classification was iteratively implemented for each combination of measured and model cutoffs. Receiver operating characteristic analysis was performed to identify the optimal cutoff. ADC, apparent diffusion coefficient; DMDR, decreased/maintained diffusion restriction; FN, false negative; FP, false positive; HGG, high-grade glioma; IDR, increased diffusion restriction; MRI, magnetic resonance imaging; RT, radiotherapy; TN, true negative; TP, true positive.

### Spatial Distribution Analysis

We analyzed the spatial distribution of IDR voxels to evaluate localization to the nonenhancing (periphery) or enhancing (core) tumor region. IDR periphery-to-core ratios were computed for measured and model data. Agreement between measured and model-predicted ratios was quantified using the concordance correlation coefficient.

### Statistical Analysis

We calculated patient-level sensitivity and specificity to assess the classification of tumor voxels as IDR or DMDR across LTCP cutoffs, with voxel-wise labels compared at matched *x*, *y*, *z* coordinates. Receiver operating characteristic (ROC) analysis was conducted to evaluate the classification performance at the cohort level and determine which LTCP cutoff provided the best correlation between categories from Δ*ADC*_*measured*_ and Δ*ADC*_*predicted*_ maps. Figure 1F describes the iterative workflow that was used to determine optimal cutoffs during ROC analysis. The AUC and Youden's index (YI) scores were used as selection criteria. When performance metrics were identical, the sensitivity for IDR was prioritized. We repeated the analysis with and without nonenhancing tumor regions at two time points (ie, *RT*_*end*_ and *RT*_*1 month*_), resulting in the following four analysis scenarios: (1) all tumor regions at *RT*_*end*_, (2) all tumor regions at *RT*_*1 month*_, (3) enhancing regions at *RT*_*end*_, and (4) enhancing regions at *RT*_*1 month*_. Dice-Sorensen coefficients (DSC) were calculated to assess spatial agreement between observed and predicted classes.

Classification performance in spatially correlated data was evaluated using bootstrap resampling (n = 1,000). In each iteration, tumor voxel coordinates were sampled with replacement, and corresponding 3 × 3 neighborhoods were extracted from both model and observed data, with edge effects handled by truncation. Sampling continued until the number of collected voxels matched or exceeded the original tumor voxel count. Voxel-wise classifications were aggregated to construct a confusion matrix, from which sensitivity and specificity were computed. ROC curves and AUC were calculated for each iteration, and overall AUC was derived from the aggregated ROC estimates. Subject-level performance is reported as mean ± standard deviation from the bootstrap analysis. Population summaries are based on the distribution of subject-level point estimates and are reported as median (IQR).

Two-tailed paired *t*-tests were performed to evaluate differences in classification performance across different forecasting time points or between data sets (with or without the nonenhancing tumor segment; voxel-level *v* neighborhood-level analysis). One-sample *t-*tests on log-transformed IDR periphery-to-core ratios assessed preferential localization relative to no bias (ratio = 1). Geometric mean (GM) ratios and 95% CIs were obtained by back-transformation from the log scale. Differences between model and measured data were evaluated using a paired *t*-test on log-transformed ratios.

## Results

Our computational framework identified tumor regions that experienced an increase in diffusion restriction and provided patient-specific spatial maps of tumor voxels categorized as IDR regions. Figure 2 reports representative results from the workflow for scenario 1 using a Δ*ADC*_*measured*_ threshold derived from an LTCP cutoff of 20%. The cases shown in Figure 2 report the worst (sensitivity: 0.76) and best (sensitivity: 0.98) performance in identifying IDR regions when forecasting treatment response at *RT*_*end*_.

**Fig 2.**
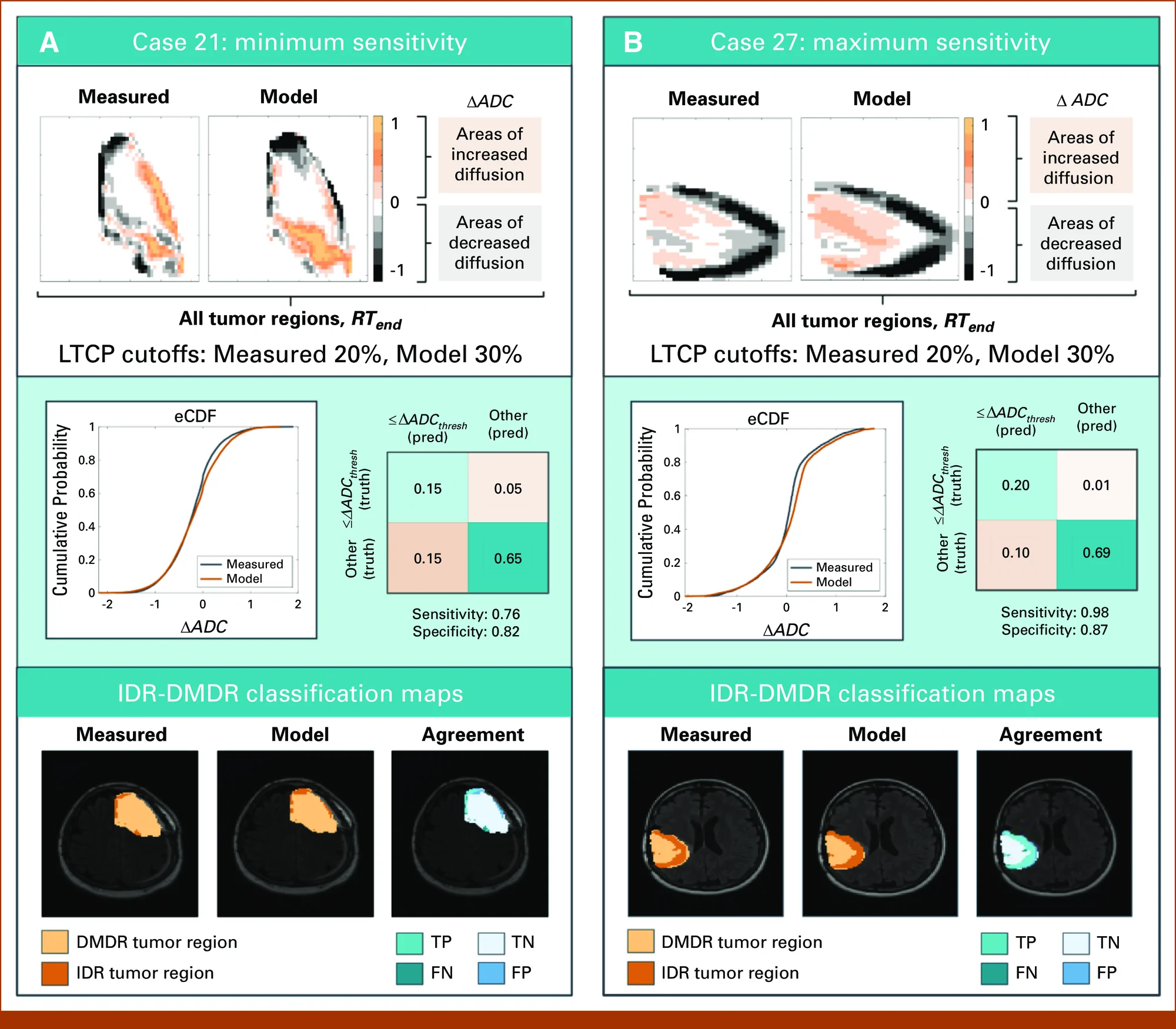
Representative results for identifying candidate biologically informed radiotherapy targets in HGG. (A) The case with minimum sensitivity for IDR regions from scenario 1 (all tumor regions at *RT*_*end*_). 2D slices of the Δ*ADC*_measured_ and Δ*ADC*_predicted_ depict areas of increased (Δ*ADC* ≥0) and decreased water diffusion (Δ*ADC* <0) shown in shades of orange and gray, respectively. Empirical cumulative distribution functions are obtained for Δ*ADC*_measured_ and Δ*ADC*_predicted_. Voxels are classified as IDR (≤Δ*ADC*_thresh_) or DMDR (other) based on LTCP cutoffs for the measured and predicted data. The overall sensitivity and specificity were 0.76 and 0.82, respectively. Voxel class labels are spatially mapped to the MRI with areas identified as DMDR and IDR regions shown in light orange and dark orange, respectively. A representative 2D slice of the measured and model classification results is presented alongside a label agreement map with TP, FN, TN, and FP depicted as light green, dark green, light blue, and dark blue areas, respectively. (B) Results for the case with maximum sensitivity. The sensitivity and specificity were 0.98 and 0.87, respectively. 2D, two-dimensional; ADC, apparent diffusion coefficient; DMDR, decreased/maintained diffusion restriction; FN, false negative; FP, false positive; HGG, high-grade glioma; IDR, increased diffusion restriction; LTCP, lower tail cumulative probability; MRI, magnetic resonance imaging; RT, radiotherapy; TN, true negative; TP, true positive.

Figure 2A presents the case with the lowest percentage of voxels correctly classified as IDR regions in the cohort. This case had 15% of the predicted tumor (2,189/14,379 voxels) correctly classified as IDR, 5% of the predicted tumor (687/14,379 voxels) incorrectly classified as DMDR, 15% (2,125/14,379 voxels) incorrectly classified as IDR, and 65% (9,378/14,379 voxels) correctly classified as DMDR. Figure 2B shows the case with highest percentage of correctly classified IDR regions in the cohort. This case had 20% of the predicted tumor (1,629/8,353 voxels) correctly identified as IDR; 1% (42/8,353 voxels) incorrectly classified as DMDR, 10% (877/8,353) incorrectly categorized as IDR, and 69% (5,805/8,353) correctly classified as DMDR tumor regions. We observe high spatial agreement of voxel labels between the measured and predicted data as shown in representative two-dimensional (2D) tumor response maps in Figure 2. Multiple comparisons of measured and model-predicted response maps (single, 2D slice visualizations) are presented in Figure 3. Representative 2D images showing IDR and DMDR areas for each subject are provided in the Data Supplement (Fig S3).

**Fig 3.**
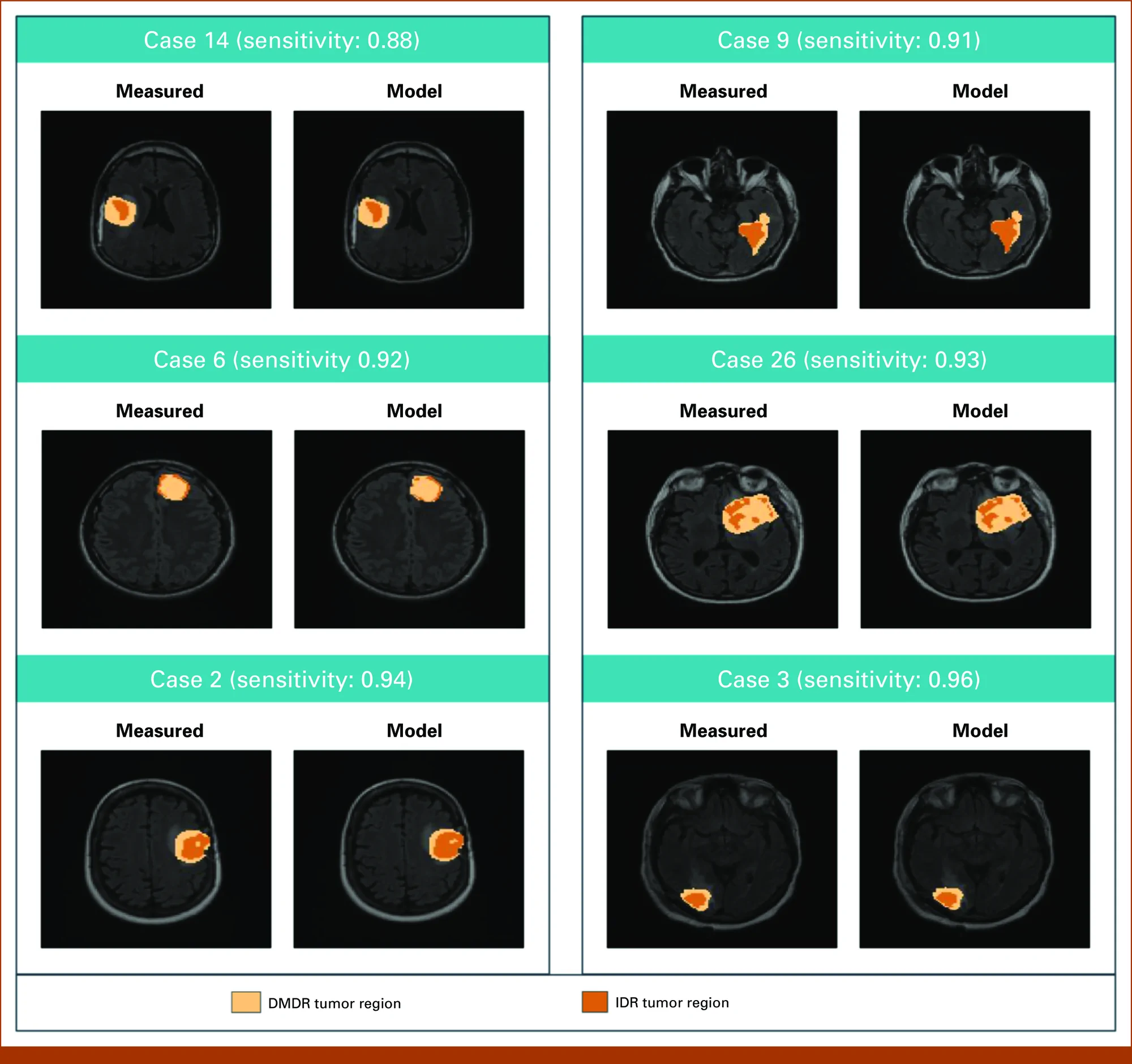
Representative results depicting 2D slices for six cases comparing areas identified as DMDR (light orange voxels) and IDR tumor areas (dark orange voxels) for the measured and model-predicted data. The cases were chosen to show the range of performance in identifying IDR tumor regions when conducting voxel-based classification of all tumor areas (enhancing, nonenhancing) at the end of radiotherapy. The analysis used Δ*ADC*_measured_ thresholds associated with an LTCP cutoff of 20% and Δ*ADC*_predicted_ thresholds at an LTCP cutoff of 30%. The results are reported in order of increasing sensitivity. The high agreement between the measured and model-predicted data demonstrates our approach can forecast, spatially locate IDR regions, and propose candidate adaptive radiotherapy targets in high-grade gliomas. 2D, two-dimensional; DMDR, decreased/maintained diffusion restriction; IDR, increased diffusion restriction; LTCP, lower tail cumulative probability.

ROC analysis established optimal LTCP cutoffs for measured and model-predicted *ADC* for all four scenarios. ROC curves for each scenario are shown in Figures 4A-4D. Across all conditions, the framework achieved high performance, with median AUC, YI, and DSC of 0.88 (range, 0.80-0.93), 0.67 (range, 0.53-0.77), and 0.88 (range, 0.73-0.95), respectively. The highest AUC for the measured data occurred at an LTCP cutoff of 20%. For model-predicted data, optimal LTCP cutoffs ranged between 30% and 35% (median 30%). ROC results, including maximum achieved AUC, YI, DSC, and optimal LTCP cutoffs for all four scenarios, are summarized in Table 1.

**Fig 4.**
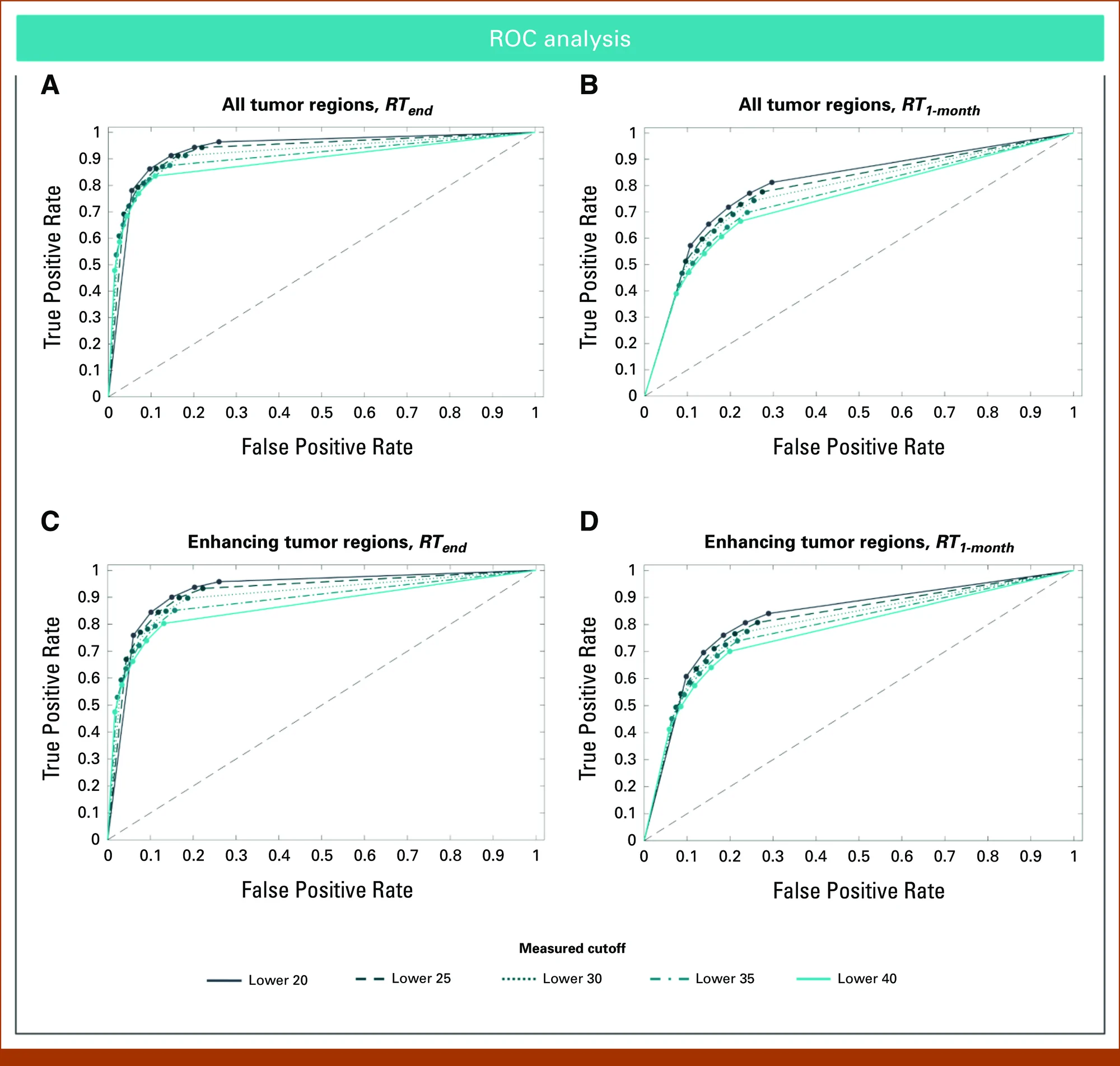
ROC analysis results used to select the optimal LTCP cutoffs. For all analysis conditions, the true-positive rates and false-positive rates shown in the diagram were calculated using the population average from the entire cohort. In each diagram, the curves represent measured LTCP cutoffs, and each point along a curve corresponds to a model LTCP cutoff. The highest AUC across all scenarios corresponded to the measured LTCP cutoff of 20% shown as a solid, black curve. (A) Scenario 1: Analyzed the entire tumor volume (contrast and noncontrast enhancing) with forecasts at the end of radiotherapy. (B) Scenario 2: Analyzed the entire tumor volume with forecasts at the 1-month follow-up visit. (C) Scenario 3: Analysis was conducted only using the contrast-enhancing tumor volume with forecasts at the end of radiotherapy; (D) Scenario 4: Analysis was conducted solely using data from the contrast-enhancing tumor volume with forecasts at the 1-month follow-up visit. In all scenarios, the computational framework is able to distinguish IDR versus DMDR tumor regions for this cohort. DMDR, decreased/maintained diffusion restriction; IDR, increased diffusion restriction; LTCP, lower tail cumulative probability; ROC, receiver operating characteristic; RT, radiotherapy.

**Table 1.** Receiver Operating Characteristic Analysis and Reports

Scenario		LTCP Cutoff		Best AUC	Youden's Index	Median DSC (range)
Number	Tumor Region, Prediction Time Point	Measured	Model			
1	All regions, *RT*_*end*_	0.20	0.30	0.93	0.77	0.87 (0.80-0.89)
2	All regions, *RT*_*1 month*_	0.20	0.35	0.80	0.53	0.76 (0.66-0.85)
3	Enhancing regions, *RT*_*end*_	0.20	0.30	0.93	0.75	0.87 (0.79-0.89)
4	Enhancing regions, *RT*_*1 month*_	0.20	0.30	0.83	0.58	0.82 (0.66-0.89)

Abbreviations: DSC, Dice-Sorensen coefficients; LTCP, lower tail cumulative probability; RT, radiotherapy.

Sensitivity and specificity at optimal LTCP cutoffs for each patient are reported in Table 2. Scenario 1 achieved a median sensitivity of 0.93 (range, 0.76-0.97) for IDR regions with a median specificity of 0.86 (range, 0.69-0.87) for DMDR regions. Median sensitivities for scenarios 2-4 were 0.78 (range, 0.53-0.99), 0.92 (range, 0.73-0.98), and 0.79 (range, 0.41-0.99), with corresponding median specificities of 0.76 (range, 0.69-0.81), 0.85 (range, 0.81-0.87), and 0.82 (range, 0.73-0.87). Spatial accuracy at optimal LTCP cutoffs is reported in the Data Supplement (Table S2). The median DSCs were 0.90 (0.82-0.93), 0.76 (0.66-0.85), 0.87 (0.79-0.89), and 0.81 (0.66-0.89) for scenarios 1-4, respectively.

**Table 2.** Summary of Voxel Classification Performance per Patient

Case Number	Sensitivity (by scenario)				Specificity (by scenario)			
	1	2	3	4	1	2	3	4
1	0.88	0.53	0.82	0.41	0.85	0.69	0.83	0.73
2	0.94	0.89	0.91	0.88	0.86	0.78	0.85	0.84
3	0.96	ND	0.93	ND	0.86	ND	0.86	ND
4	0.95	0.95	0.95	0.84	0.86	0.80	0.86	0.84
5	0.96	0.80	0.95	0.83	0.86	0.76	0.86	0.83
6	0.92	0.57	0.87	0.54	0.86	0.71	0.84	0.76
8	0.82	0.73	0.74	0.80	0.83	0.75	0.81	0.82
9	0.90	0.99	0.98	0.99	0.85	0.81	0.87	0.87
10	0.95	0.87	0.94	0.94	0.86	0.78	0.86	0.86
11	0.91	0.96	0.92	0.93	0.85	0.80	0.85	0.86
14	0.88	0.78	0.93	0.96	0.85	0.76	0.86	0.87
16	0.96	0.76	0.96	0.75	0.87	0.75	0.87	0.81
17	0.90	0.80	0.87	0.78	0.85	0.76	0.84	0.82
18	0.94	0.94	0.93	0.88	0.86	0.80	0.86	0.85
20	0.84	0.61	0.83	0.53	0.84	0.72	0.83	0.76
21	0.76	0.77	0.73	0.68	0.82	0.76	0.81	0.79
23	0.90	0.79	0.92	0.74	0.85	0.76	0.85	0.81
24	0.93	0.72	0.90	0.67	0.86	0.74	0.85	0.79
26	0.93	0.64	0.91	0.66	0.86	0.72	0.85	0.79
27	0.97	0.74	0.98	0.79	0.87	0.75	0.87	0.82
28	0.93	0.57	0.92	0.59	0.86	0.70	0.85	0.77
Median	0.93	0.78	0.92	0.79	0.86	0.76	0.85	0.82
Minimum	0.76	0.53	0.73	0.41	0.82	0.69	0.81	0.73
Maximum	0.97	0.99	0.98	0.99	0.87	0.81	0.87	0.87

Abbreviations: ND, no data; sensitivity, sensitivity for IDR regions using the optimal LTCP cutoffs; specificity, specificity for DMDR regions using the optimal LTCP cutoffs.

No statistically significant differences in classification accuracy and spatial agreement were observed when comparing performance between forecasting time points (*P* values for AUC: .08, YI: .10, DSC: .24). Including or excluding the nonenhancing tumor volume did not significantly affect AUC (*P* value: .50), YI (*P* value: .74), or DSC (*P* value: .53) when comparing scenario 1 with scenario 3 and scenario 2 with scenario 4. The workflow performed best in scenario 1 (AUC: .93, YI: .77, DSC: .87).

Measured and model-predicted IDR periphery-to-core ratios showed high concordance (CCC: 0.90), and no significant difference was observed between log-transformed ratios (paired *t*-test, *P* = .052). Both data sets exhibited tumor core localization (measured: GM, 0.14 [95% CI, 0.07 to 0.28], *P* value: 7.33 × 10^−6^; model: GM, 0.23 [95% CI, 0.16 to 0.36], *P* value: 5.15 × 10^−7^), as shown by subject-wise periphery-to-core ratio comparisons and observed versus predicted ratios in the Data Supplement (Fig S4). When neighboring voxels were treated as spatially correlated, bootstrap analysis yielded population-level AUCs of 0.80 (IQR: 0.02), 0.69 (IQR: 0.01), 0.80 (IQR: 0.01), and 0.74 (IQR: 0.11) for scenarios 1-4, respectively. Population-level sensitivity and specificity, along with subject-level performance metrics, are presented in the Data Supplement (Table S3). Voxel-level classification significantly outperformed (*P* value: .001) the neighborhood-based approach.

## Discussion

Our results demonstrate that biologically informed RT targets, characterized by an increase in tumor cell density approximated from *ADC* changes following treatment, can be reliably identified. Using a computational framework combining biology-informed mathematical modeling and statistical analysis, IDR regions (ie, areas with increased tumor cell density following treatment) were detected with high sensitivity (ie, 0.93, 0.78, 0.92, and 0.79 for scenarios 1-4, respectively). This approach enables patient-specific prediction of tumor response and supports RT personalization based on anticipated biological changes. Detected IDR regions may provide a basis for biologically guided intratumoral dose modulation, with candidate target volumes defined by forecasted intratumoral heterogeneity in Δ*ADC* rather than geometric criteria or anatomical constraints.

Our findings show that voxel-level assessment of changes in diffusion restriction captures regional intratumoral differences, with IDR regions present in both tumor core and periphery across patients. Measured and model-predicted IDR periphery-to-core ratios showed high concordance (CCC: 0.90), with no significant difference between log-transformed ratios, indicating similar spatial distribution patterns in both data sets. Despite interpatient variability, IDR regions showed preferential localization to the tumor core, aligning with sites of reported RT failure^14^ and contrasting with the periphery-focused targets common in adaptive RT strategies. This framework provides spatially resolved identification of candidate intratumoral regions of interest, which may inform future adaptive RT strategies.

This framework extends prior approaches that define RT targets by expanding the contrast-enhancing lesion using fixed or variable margins^9-16,40,41^ or by modeling outward tumor propagation,^40,42^ with or without anatomical constraints.^10,43^ In contrast, our method defines candidate regions based on anticipated growth patterns and intratumoral biological heterogeneity. It can be applied as an alternative or a complement to existing techniques: as an alternative, it supports prediction of tumor growth and delineates candidate subregions for adaptive dose escalation within the predicted volume; as a complement, it refines candidate target selection both within and beyond this volume when combined with margin-based boost strategies. In both cases, the framework captures intratumoral heterogeneity (Δ*ADC*), offering a biologically informed basis for identifying candidate regions for adaptive RT.

This study identifies several avenues for further improvement and investigation. First, the study should be validated in a larger cohort beyond the current sample size (N = 21). Second, this framework should be evaluated on external data sets across multiple institutions to assess the flexibility of the method to variations in DWI protocols, differences in image acquisition parameters affecting *ADC* measurement, and data quality controls which may vary per institution. Third, the cascaded nature of our forecasting and spatial mapping algorithms inherently propagates forecasting errors into the data-driven approach to identify RT targets. Incorporating approaches to quantify uncertainty and error at each data processing step (ie, image acquisition, registration, segmentation) and modeling stage (ie, forecasting and spatial mapping) could potentially improve future iterations of this work.

Moreover, *ADC* values are influenced by multiple biological and treatment-related factors—including hemorrhage, infarction, inflammation, and pseudo-progression—limiting the biological specificity of diffusion-based metrics and, by extension, our *ADC*-based framework. The absence of perfusion imaging further limits the framework's ability to differentiate viable tumor from nontumor regions exhibiting restricted diffusion. Future work could address this by incorporating additional MRI metrics, such as susceptibility-weighted imaging to account for hemorrhage and perfusion imaging to better characterize tumor properties. Additionally, the IDR-DMDR classification cannot be directly validated through histopathology and should be interpreted as an exploratory imaging biomarker pending further validation. As diffusion-based models continue to evolve, this framework offers a foundation for developing more comprehensive multiparametric models for personalized tumor forecasting and adaptive RT regimens.

In conclusion, we developed a computational framework for forecasting and spatially mapping patient-specific candidate RT targets in HGG. This provides a biologically informed basis for investigating intratumoral dose modulation strategies in adaptive RT.

## Data Availability

A data sharing statement provided by the authors is available with this article at DOI https://doi.org/10.1200/CCI-25-00336.
